# MLK4 promotes glucose metabolism in lung adenocarcinoma through CREB-mediated activation of phosphoenolpyruvate carboxykinase and is regulated by KLF5

**DOI:** 10.1038/s41389-023-00478-y

**Published:** 2023-07-05

**Authors:** Alvin Ho-Kwan Cheung, Kit-Yee Wong, Xiaoli Liu, Fenfen Ji, Chris Ho-Lam Hui, Yihan Zhang, Johnny Sheung-Him Kwan, Bonan Chen, Yujuan Dong, Raymond Wai-Ming Lung, Jun Yu, Kwok Wai Lo, Chi Chun Wong, Wei Kang, Ka-Fai To

**Affiliations:** 1 Department of Anatomical and Cellular Pathology, State Key Laboratory of Translational Oncology, Prince of Wales Hospital, The Chinese University of Hong Kong, Shatin, Hong Kong; 2 Institute of Digestive Disease, Department of Medicine and Therapeutics, State Key Laboratory of Digestive Disease, Li Ka Shing Institute of Health Sciences, The Chinese University of Hong Kong, Hong Kong SAR, China

**Keywords:** Lung cancer, Cancer metabolism

## Abstract

MLK4, a member of the mitogen-activated protein kinase kinase kinase (MAP3K) family, has been implicated in cancer progression. However, its role in lung adenocarcinoma has not been characterized. Here, we showed that MLK4 was overexpressed in a significant subset of lung adenocarcinoma, associated with a worse prognosis, and exerted an oncogenic function in vitro and in vivo. Bioinformatics analyses of clinical datasets identified phosphoenolpyruvate carboxykinase 1 (PCK1) as a novel target of MLK4. We validated that MLK4 regulated PCK1 expression at transcriptional level, by phosphorylating the transcription factor CREB, which in turn mediated PCK1 expression. We further demonstrated that PCK1 is an oncogenic factor in lung adenocarcinoma. Given the importance of PCK1 in the regulation of cellular metabolism, we next deciphered the metabolic effects of MLK4. Metabolic and mass spectrometry analyses showed that MLK4 knockdown led to significant reduction of glycolysis and decreased levels of glycolytic pathway metabolites including phosphoenolpyruvate and lactate. Finally, the promoter analysis of MLK4 unravelled a binding site of transcription factor KLF5, which in turn, positively regulated MLK4 expression in lung adenocarcinoma. In summary, we have revealed a KLF5-MLK4-PCK1 signalling pathway involved in lung tumorigenesis and established an unusual link between MAP3K signalling and cancer metabolism.

## Introduction

Lung cancer is the leading cause of cancer death worldwide in men, and second in women after breast cancer [1]. Adenocarcinoma accounts for about 35% of all lung cancer [2]. With the advent of sequencing technologies, many driver mutations and chromosomal translocations of lung adenocarcinoma are now known and amenable to targeted therapy [3]. However, despite efforts in developing new treatments, lung adenocarcinoma can have an aggressive clinical course and results in major patient morbidity and mortality [4].

Among cancers, lung adenocarcinoma has a relatively high mutation load [5, 6], implying additional genetic aberrations may account for its aggressiveness in addition to known driver mutations. A recent study comprehensively sequenced paired primary and metastatic cancer tissue, including 234 lung cancer patients, and identified *MLK4* as one of the important genes which may play a role in cancer progression and metastasis [6]. However, for lung adenocarcinoma, the functional role of MLK4, its downstream targets, as well as its regulation remain largely unexplored in the literature.

MLK4 is a relatively poorly characterized serine/threonine kinase, and belongs to the mixed lineage kinase (MLK) family of MAP3K [7]. The classes of MAP3K kinases mediate the classical signal transduction pathway to regulate cell differentiation and cell cycle, in concert with ERK1/2. Other downstream signaling components of MLK4 include JNK, p38, and NF-κB cascades [8, 9]. As diverse pathways are involved, it might not be surprising to observe contrasting oncogenic and tumour suppressive functions of MLK4 in different cancer types. In breast cancer [10, 11] and glioma [12], MLK4 exerts its oncogenic function by epithelial-mesenchymal transition activation and NF-κB activation, respectively. MLK4 aberrations were shown to be associated with oncogenic *KRAS* signaling in colorectal cancer [13], yet there were also reports that MLK4 could suppress ovarian [14] and colorectal [15] carcinogenesis. Recently, the kinase was also shown to influence DNA damage repair and promote cancer cell chemoresistance[16].

In this study, we demonstrate that *MLK4* gene overexpression serves as an oncogenic event in lung adenocarcinoma, and dissect the role of the transcription factor KLF5 in driving MLK4 regulation. Downstream to MLK4, we also probed into other possible downstream effectors by bioinformatics and RNA-sequencing analyses, and unexpectedly found an association of MLK4 kinase activity to lung cancer metabolism. This link between cell cycle signaling pathway and glucose metabolism was unusual, and involved phosphoenolpyruvate carboxykinase 1 (PCK1), an enzyme catalyzing the rate limiting step of oxaloacetate conversion to phosphoenolpyruvate (PEP) in the process of gluconeogenesis [17, 18]. Together with the findings that KLF5, MLK4, and PCK1 appeared to be correlated in their expressions and predict a worse prognosis in lung adenocarcinoma, the study proposed a model of KLF5-MLK4-PCK1 signaling cascade that promotes glucose metabolism and tumorigenesis.

## Materials and methods

### Cell and organoid culture

Lung adenocarcinoma cell lines A-549 (RRID: CVCL_0023), NCI-H23 (RRID: CVCL_1547), NCI-H358 (RRID: CVCL_1559), NCI-H1792 (RRID: CVCL_1495), NCI-H2030 (RRID: CVCL_1517), NCI-H1563 (RRID: CVCL_1475), NCI-H1975 (RRID: CVCL_1511), NCI-H3255 (RRID: CVCL_6831), NCI-H1650 (RRID: CVCL_1483), and NL20 were obtained from American Type Cell Culture, and were maintained in appropriate media according to cell line provider’s instructions. All human cell lines have been authenticated using STR profiling within the last three years. Low glucose culture media were prepared by supplementing the base DMEM or RPMI1640 media with 1 mM D-glucose and 10 mM L-lactate. Patient-derived organoids were produced by dissecting the tumour samples and digesting the dissected tissue in collagenase-digestion buffer, and resuspending into Matrigel matrix (356231, Corning) for 30 min. After the matrigel matrix was polymerized, lung cancer specific organoid culture media containing supplements were added and the dish was placed in an incubator with a temperature of 37 °C, under 5% CO_2_ humidity. All experiments were performed with mycoplasma-free cells. The study was approved by ethics committee of the Chinese University of Hong Kong.

### Immunohistochemistry and clinical samples

Immunohistochemistry (IHC) was carried out on tissue microarray using Benchmark XT autostainer (Ventana, Tucson, AZ) using Ultraview detection system, with monoclonal antibody against MLK4 (ab93798, Abcam), PCK1 (ab133603, Abcam), and KLF5 (ab137676, Abcam). The tissue microarray was generated from a cohort of 166 lung adenocarcinoma patients from year 1995 to 2010, at the Prince of Wales Hospital, Hong Kong. The formalin-fixed paraffin-embedded tissues were used in this study. Assessment of the immunohistochemical findings were made by pathologists blinded to the nature of the samples.

### Cell transfection and in vitro functional studies

All transfection assays with siRNAs were performed using Lipofectamine™ 2000 Transfection Reagent (Invitrogen). The pharmacologic agents K252a, URMC-099, and 666-15 were obtained from Cell signaling (#12754), and Selleck Chem (S7343, S8846). All siRNA against MLK4, PCK1, PCK2, CREB and KLF5 were obtained from Qiagen. Transfection of the plasmid for the overexpression of MLK4 was carried out with the FuGene HD transfection reagent (Promega). The MLK4 expression plasmid was based on a pcDNA3.1(+) backbone, with the insert being the full length of the MLK4 coding sequence. To generate the kinase dead MLK4 plasmid, a mutation of the kinase domain of MLK4 was introduced to the MLK4 expression plasmid by means of site-directed mutagenesis, using the QuikChange II Site-directed mutagenesis kit (Agilent) according to the manufacturer’s instructions. Knockdown of MLK4 in cancer cells were carried out by transduction of lentivirus packaged with the LentiCRISPRv2 vector (Addgene, #52961) cloned with sgRNAs, detailed in Supplementary Table 1, followed by single clone selection with puromycin. The Cell proliferation was assessed using CellTiter 96 Non-Radioactive Cell Proliferation Assay (Promega, Madison, WI). Colony formation assays was performed by seeding the transfected cells in a 6-well plate and incubated for 7–10 days, and colonies counted with 0.2% crystal violet staining. Cell invasion assays (354480, Corning) were performed as per the manufacturer’s instructions. Apoptosis assay was performed using the FITC Annexin V Apoptosis Detection Kit I (BD, Franklin Lakes, NJ). Cell cycle analysis was performed by staining with propidium iodide (Sigma-Aldrich) after fixation of cells in 70% ethanol overnight. The subsequent flow cytometry was performed on BD LSRFortessa cell analyser.

### Quantitative polymerase chain reaction

Total RNA extraction was performed with the Trizol reagent. Reverse transcription was performed with PrimeScript RT reagent kit (Takara). Real time PCR were performed using the Quantstudio Flex 1 real time PCR system (ThermoFisher), with the TB Green Premix Ex Tag kit (Takara). The primer sequences used in this study were listed in Supplementary Table 1.

### Protein extraction, Western blot analysis, and immunofluorescence study

Protein lysate was obtained by cell lysis in the RIPA buffer, and subsequently resolved by sodium dodecyl sulfate-polyacrylamide gel electrophoresis. Transfer to polyvinylidene difluoride membranes (GE Healthcare, Piscataway, NJ) was carried out. The antibodies against Phospho-MEK1/2 (#9121), MEK (#9122), p21 (#2946), p27 (#2552), Phospho-Rb (Ser807/811) (#9308), cleaved PARP (Asp214) (#9541), PARP (#9542), CREB (#4820), phospho-CREB for phosphorylation of Ser133 (#9198), were obtained from Cell signaling (Danvers, MA). Antibodies against MLK4 (ab93798), KLF5 (ab137676), PCK1 (ab133603), PCK2 (ab187145), were obtained from Abcam. Antibody against BCL2 (YM3041) was obtained from Immunoway (TX, US). GAPDH (#2118, Cell signaling) expression was used as the equal loading control. Probing of the membrane with the primary antibodies was performed at 4 °C overnight, and with horseradish peroxidase-conjugated secondary antibody at room temperature for 1 h. The membranes were developed and captured on the ChemiDoc XRS machine (Bio-Rad) using ImageLab software.

Primary antibodies for immunofluorescence study included MLK4, pCREB, and CREB. Counterstaining of cell nuclei was performed with DAPI (Sigma-Aldrich). Images were acquired with a confocal microscope, model Carl Zeiss LSM880 (Gottingen, Germany).

### Metabolism studies

For Liquid chromatography-mass spectrometry (LC-MS) of intracellular metabolites, cells were washed by PBS three times, and then homogenized in 500 μl of cold methanol/acetonitrile/water (4:4:2) mixture solution containing 0.1% formic acid and internal standard 4Cl-phenylalanine by using a Polytron PT2100 homogenizer. Subsequently the cells were subjected to 3 freeze-thaw cycles under liquid nitrogen. After centrifugation at 21,500 × *g* for 10 min at 4 °C, the supernatant was dried under vacuum and the cell pellet was dissolved in 0.1 N NaOH for protein determination and further metabolite normalization. The dried extract was reconstituted in 100 μl of 80% methanol. 20 μl of the solution was taken out and derivatized by 3-nitrophenylhydrazine as appropriate for analysis. Instrument analysis was carried on a Thermo Scientific UPLC system coupled to a TSQ Quantiva™ Triple Quadrupole MS equipped with an ESI source. A Waters BEH amide analytical column (1.7 μm, 2.1 mm × 100 mm) was employed as appropriate, being kept at 40 °C during the analysis. The mobile phase consisted of water/acetonitrile (95:5) with 20 mM ammonium acetate and 20 mM ammonium hydroxide and acetonitrile. Derivatized metabolites were separated by a Waters HSS T3 column (1.8 μm, 2.1 mm × 100 mm) under 40 °C. The mobile phases were water containing 0.01% FA and acetonitrile. Mass spectrometry analysis was both performed in negative ion multiple reaction monitoring mode. Metabolite signals were normalized to internal standard and protein content for cell pellet. For the determination of glycolysis and mitochondrial respiration, the Seahorse XF Glycolytic Rate Assay Kit (Agilent) and the Seahorse XF Cell Mito Stress Test Kit (Agilent) were used respectively, on the Seahorse XFe96 cell analyser instrument (Agilent), according to manufacturer’s instructions. The investigation of PEPCK enzymatic activity was performed by using the PEPCK activity assay (ab239714, Abcam), performed according to manufacturer’s protocols.

### Immunoprecipitations and ChIP-qPCR

Following cell culture and fixation of samples, cross-linked chromatin were sheared by Covaris sonication system (S220) to desired fragment lengths of 300–500 base pairs. The sonicated products were incubated with Magnetic Dynabeads Protein G (1004, Life Tech) and linked with anti-KLF5 (ab137676, AbCam), anti-CREB antibody (#4820, Cell signaling), or Normal anti-IgG antibody (#2729, Cell signaling). ChIP-qPCR was performed using primers binding to the putative binding sites of MLK4 and PCK1, in order to calculate the relative enrichment of amplicons between the target antibody and anti-IgG antibody.

### Luciferase activity assays

The putative transcription factor binding site at the promoter of MLK4 was sub-cloned into the pGL3-Basic vector (Promega). The sequences of inserts for the vector containing the MLK4 promoter and the control vector were detailed in Supplementary Table 1. The detection of luciferase reporter activity was subsequently measured with the Dual-Luciferase Reporter Assay System (Promega) according to the assay protocol.

### Bioinformatics analysis

The cancer genome atlas (TCGA) clinical data for lung adenocarcinoma patients in this study was analyzed on the cBioportal database. Gene set enrichment analysis was performed with gene sets detailed in the MSigDB database. Gene ontology analysis was carried out to identify enriched gene set using the R package ‘clusterprofiler’ (version 3.18). For single cell sequencing analysis, further analysis was performed on the lung adenocarcinoma single cell sequencing dataset GSE131907 on publicly available GEO repository. The R package ‘Seurat’ (version 4.0.2) was used to perform dimension reduction with t-distributed stochastic neighbor embedding analysis, cell clustering, and visualization.

### Animal studies and xenograft models

Xenografts were produced by injecting A549 cancer cells (1 × 10^6^ cells/mouse, diluted in 0.1 ml PBS) subcutaneously into the dorsal flank of Balb/c NOD-SCID mice at the same time for each group. Sample size estimate and blinding were not applicable to this animal study. The tumour size was measured every 3 days. The mice were sacrificed 3 weeks after inoculation and the xenografts were harvested. For the orthotopic lung implantation model, cancer cells were injected into the mice through the intrathoracic route to induce tumour formation. The xenograft was monitored with bioluminescence imaging. All animal work was approved by the Animal Experimentation Ethics Committee of the Chinese University of Hong Kong.

### Statistical analyses

Continuous variables were expressed as means ± SD. Treatment groups were compared with the two-tailed independent or paired sample *t*-test where appropriate. Data were acquired from three independent experiments unless otherwise specified. *p*-values < 0.05 were considered statistically significant. Error bars indicated the standard deviation. Survival difference was studied using the log-rank statistics. Analyses were performed with the SPSS statistics software (version 20, SPSS Inc., Chicago, IL) and the Graphpad Prism software (version 8.0.1).

## Results

### MLK4 alteration occurs in a significant subset of lung adenocarcinoma and portends a worse prognosis

**Fig. 1:**
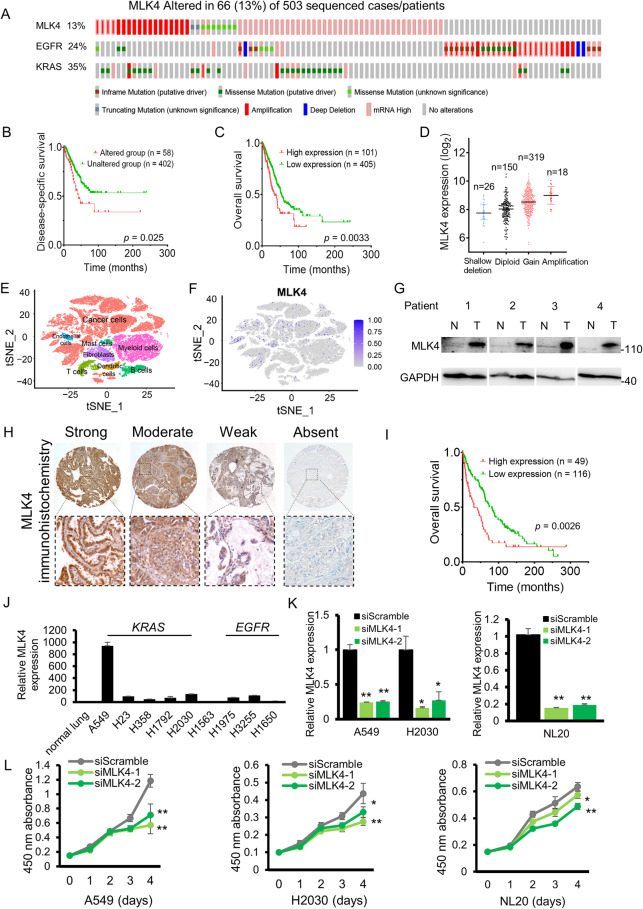
MLK4 is significantly overexpressed in lung adenocarcinoma and correlated with worse survival. **A** Genetic and chromosomal alterations of MLK4 in lung adenocarcinoma patients from the Cancer Genome Atlas (TCGA) PanCancer Atlas cohort from cBioportal. Other TCGA cohorts from cBioportal may contain overlapping samples and were analyzed separately in Supplementary Fig. 1. **B**, **C** (**B**) Alteration and (**C**) upregulation of MLK4 portended a worse survival in TCGA patient cohort. **D** The correlation between MLK4 mRNA expression and its copy number alterations (including shallow deletion, diploid, gain and amplification). **E** Single cell RNA-seq analysis revealed cancer cells and supporting cells in the microenvironment as constituents of the tumours. **F** Upregulation of MLK4 was observed in cancer cells predominantly from single-cell RNA-seq data. **G** Western blot showing increased expression of MLK4 in patient lung adenocarcinoma samples (labeled T), compared to adjacent normal lung tissue (labeled N). **H** Immunohistochemistry (IHC) of MLK4 in lung adenocarcinoma tissue microarray, showing predominantly cytoplasmic staining in cancer cells. 40× (above) and 100× magnification (below). **I** MLK4 protein overexpression, as indicated by strong or moderate MLK4 IHC staining, was associated with a worse clinical outcome. **J** MLK4 expression in *KRAS*-mutated (A549 through H2030) and *EGFR*-mutated (H1975 through H1650) cell lines was generally higher compared to normal. **K** The mRNA expression of MLK4 after siRNA-mediated knockdown in A549 and H2030 cells, and the normal lung cell line, NL20. **L** siRNA-mediated knockdown of MLK4 inhibited cancer cell proliferation, and to the lesser extent in NL20 (*p < 0.05; **p < 0.005).

We observed that *MLK4* alteration was a common occurrence in lung adenocarcinoma, from The Cancer Genome Atlas (TCGA) cohort (Fig. 1A). Other cohorts from cbioportal also showed a significant occurrence of *MLK4* alterations (Supplementary Fig. 1). Upregulation and amplifications are the most common forms of *MLK4* aberrations, and these can be present together with other driver mutations such as *EGFR* and *KRAS* mutations (*p* > 0.05 for tests of mutual-exclusivity). Patients with any *MLK4* alterations that were indicated in Fig. 1A or overexpression in RNA (Fig. 1B, C) could have a worse survival clinically. Overexpression of MLK4 in lung adenocarcinoma could be due to a low-level copy number gain or amplification (Fig. 1D). To understand whether the observed MLK4 overexpression in tumour tissue was due to cancer cells or other supporting cells in the tumour microenvironment, we analyzed the available single cell RNA sequencing dataset for lung cancer. A diversity of cell types were present in the tumour tissue other than cancer cells, such as fibroblasts, immune cells and endothelial cells (Fig. 1E). MLK4 overexpression was largely detected in the cancer cells (Fig. 1F), suggesting that MLK4 overexpression may be an oncogenic event rather than a reactive phenomenon of other supporting cells. Overexpression of MLK4 could be observed in patient lung adenocarcinoma samples compared to adjacent normal lung tissue (Fig. 1G). Immunohistochemical studies on tissue microarray revealed that the staining of MLK4 was predominantly localized in the cytoplasm, and a spectrum of staining intensity was observed (Fig. 1H). Lung adenocarcinoma with high or moderate MLK4 staining intensity accounted for about 30% of the patients, and these patients had a significantly worse prognosis (Fig. 1I).

### MLK4 upregulation is an oncogenic event in lung adenocarcinoma

To investigate the functional role of MLK4 in lung adenocarcinoma, we performed knockdown of MLK4 to examine its effect on the tumour cells in vitro. MLK4 is overexpressed in many lung adenocarcinoma cell lines, with the highest expression noted for A549, H2030, and H3255 (Fig. 1J). At this juncture, it was decided to select the *KRAS*-mutated cell lines A549 and H2030 for further investigation, firstly because *KRAS* mutation is the most common driver mutation in the Caucasian population, accounting for about 20–40% of all cases [19, 20], and secondly because the overexpression of MLK4 appeared to be more prominent in *KRAS*-mutated cell lines compared to *EGFR*-mutated cell lines (Fig. 1J). After knockdown of MLK4 with siRNA (Fig. 1K), the cell proliferation of cancer cells were decreased (Fig. 1L). As a control, the NL20 normal lung cells were employed for the knockdown assays as well (Fig. 1K), and it also demonstrated a decreased cell proliferation on MLK4 knockdown, although to a lesser extent compared to the cancer cells (Fig. 1L). This is in keeping with the markedly lower expression of MLK4 in NL20 compared to the cancerous cell lines.

**Fig. 2:**
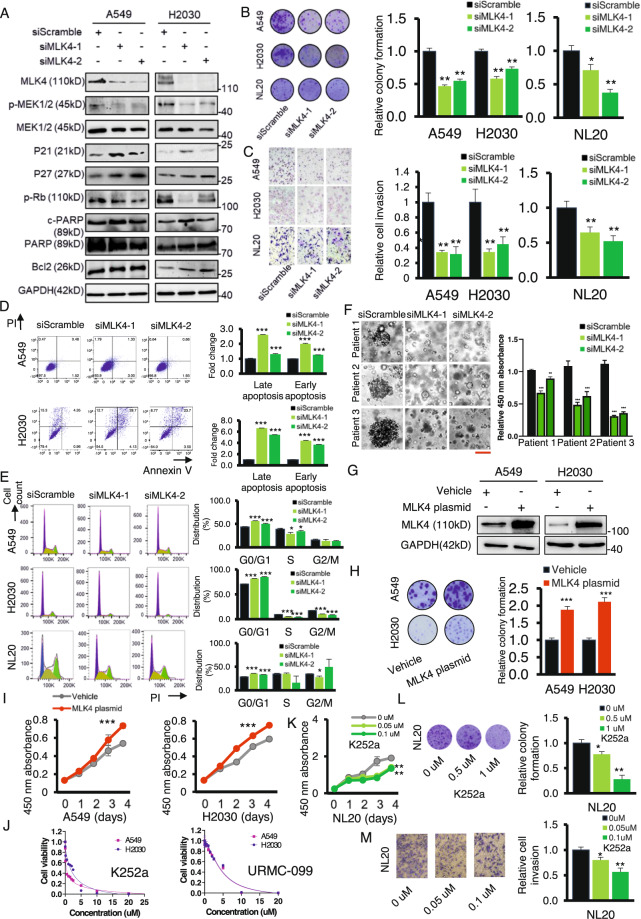
Depletion of MLK4 exerted an anti-tumour effect in vitro. **A** Western blot analysis of the cell cycle signaling and apoptosis biomarkers after MLK4 knockdown. **B** Knocking down MLK4 resulted in a reduction of monolayer colony formation ability of the cancer cells and normal lung control (*p < 0.05; **p < 0.005). **C** MLK4 knockdown inhibited cell invasion ability in transwell matrigel invasion assay (*p < 0.05). **D** siMLK4 transfectants exhibited a higher cell subpopulation in the early and late apoptosis as demonstrated by flow cytometry (***p < 0.0005). **E** FACS analysis for the cell cycle distribution in lung cancer and normal cells with and without knockdown of MLK4. (***p < 0.0005) **F** Representative photomicrographs and cell viability of patient-derived organoids treated with siMLK4. Scale bar, 50 μm. (*p < 0.05; **p < 0.005; ***p < 0.0005). **G** Western blot analyses for the overexpression of MLK4 in cell lines. **H** MLK4 overexpressed transfectants demonstrated an increased in colony formation ability. **I** MLK4 overexpression led to an increased cancer cell proliferation. (*p < 0.05; **p < 0.005; ***p < 0.0005). **J** Inhibition of cancer cells with the first and newer generation of mixed lineage kinase inhibitor, K252a and URMC-099 respectively, in micromolar concentration. **K**–**M** The effect of the inhibitor K252a on normal lung cell line, NL20. (*p < 0.05; **p < 0.005), in terms of (**K**) cell proliferation, (**L**) colony formation ability, (**M**) and the ability to migrate through matrigel wells.

When knockdown of MLK4 was performed, the expression of proteins related to cell cycle arrest, including the cyclin-dependent kinase inhibitors p21 and p27, were generally increased (Fig. 2A). Whereas the expression of proteins related to cell cycle progression, such as phosphorylated retinoblastoma protein (pRb) was decreased; while those related to apoptosis, such as cleaved-PARP, was induced (Fig. 2A). The MEK1/2 protein, the well-known downstream target of MLK4, was also inhibited in terms of its phosphorylation by MLK4 knockdown (Fig. 2A). Knockdown of MLK4 by siRNA showed that the colony formation ability of the cell lines were consistently decreased (Fig. 2B). The invasion abilities of the cell lines were also decreased (Fig. 2C). The inhibitory effect of MLK4 knockdown could also be accounted for by increased cell apoptosis (Fig. 2D), as well as by decreased cell cycle progression (Fig. 2E). The inhibitory effect on tumour cell growth was further evidenced by MLK4 knockdown in patient-derived organoids (Fig. 2F). Conversely, overexpression of MLK4 in cancer cells (Fig. 2G) resulted in an increase in colony formation ability (Fig. 2H) and cell proliferation (Fig. 2I). Because specific pharmacological inhibitors for MLK4 are lacking, we had to select the first generation MLK inhibitor K252a and the newer class of more selective MLK inhibitor URMC-099 to examine the effect of these inhibitors on cancer cells. These inhibitors inhibited both cell lines in micromolar concentration (Fig. 2J), while its effects on normal lung cells were shown in Fig. 2K–M and Supplementary Fig. S2. K252a was selected because it was a prototypical drug which had been modified to give further MLK inhibitor and the new inhibitor URMC-099 was chosen for its distinct chemical structure from K252a and the ability to cross blood-brain barrier which may be promising to treat metastatic tumour.

### MLK4 regulates the transcription of PCK1 through phosphorylation of CREB

**Fig. 3:**
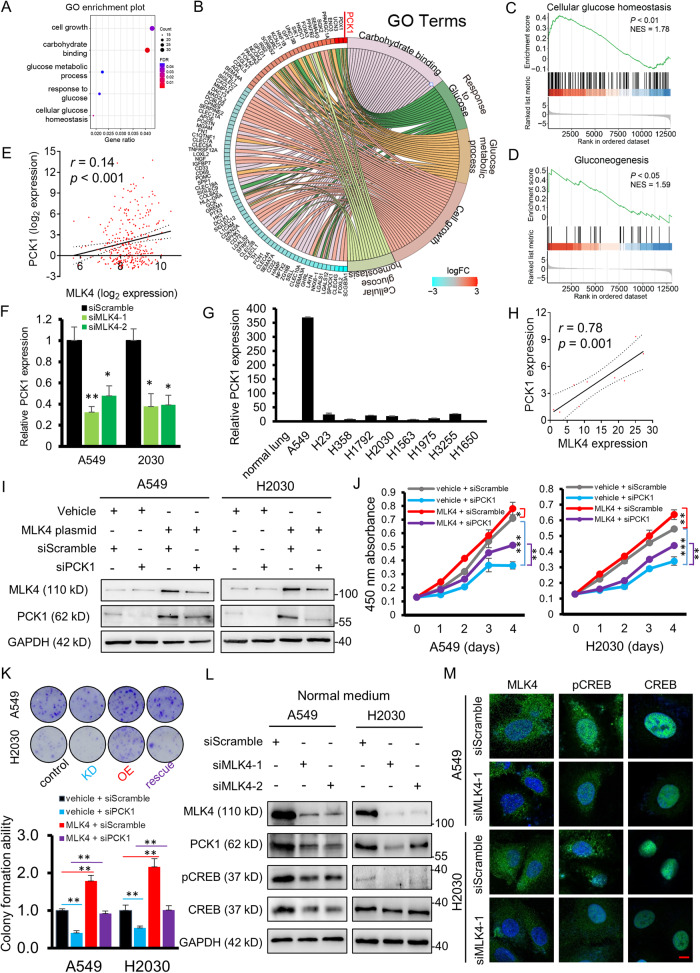
PCK1 is a downstream molecule of MLK4 and is transcriptionally regulated by MLK4. **A** Gene ontology (GO) analysis of patients stratified into high and low MLK4 expression (see text) in the Cancer Genome Atlas (TCGA) cohort enriched glucose metabolism related pathways. **B** Genes with significantly altered expression levels within the gene set enriched, where PCK1 was identified. **C**, **D** Gene set enrichment analysis GSEA demonstrated a positive correlation between MLK4 upregulation and glucose metabolism related processes. NES, normalized enrichment score. **E** MLK4 mRNA expression showed a tendency to correlate with that of PCK1 in the TCGA cohort. **F** Knockdown of MLK4 resulted in a reduction in mRNA expression in PCK1 (*p < 0.05; **p < 0.005). **G** PCK1 mRNA expression in KRAS-mutated (A549 through H2030) and EGFR-mutated (H1975 through H1650) cell lines. **H** Correlation of MLK4 and PCK1 mRNA expression in cell lines. The expression of MLK4 and PCK1 in A549 were both excessively high compared to other cell lines, and thus was not shown in this diagram. **I** Western blot analysis of rescue experiments performed in A549 and H2030 cell lines. **J**, **K** Rescue experiments showed that the inhibitory effect on (**J**) cell proliferation and (**K**) monolayer colony formation by PCK1 knockdown in cancer cells could be rescued by MLK4 overexpression. Groups denoted in (**K**): Control: Vehicle + siScramble; Knockdown (KD): Vehicle + siPCK1; Overexpressed (OE): MLK4 plasmid + siScramble; rescue: MLK4 plasmid + siPCK1 (**p < 0.005; ***p < 0.0005). **L** Western blot analyses demonstrated that MLK4 knockdown caused a reduction of phosphorylated form of the transcription factor CREB and the expression of PCK1 in normal supplemented medium. **M** Representative photomicrographs of immunofluorescence images showing the expression of targeted proteins in green and DAPI in blue. Scale bar, 10 μm.

Subsequently, we decided to explore what other potential downstream targets of MLK4 may be implicated in lung adenocarcinoma. To this end, we decided to perform pathway analyses on TCGA RNA expression data generated from human lung adenocarcinoma samples. By stratifying tumours into those having the highest MLK4 expression (highest 10% of all cases) and the lowest MLK4 expression (lowest 10% of all cases), we examined the difference in the transcriptome and the pathways which were associated with a higher level of MLK4 expression. By gene ontology (GO) analysis, we found that pathways pertaining to glucose metabolism were significantly enriched (Fig. 3A). When we arranged the genes within these pathways by their RNA expression, the gene ranked highest in the set was found to be PCK1, which was related to the response to glucose, glucose metabolic process and cellular glucose homeostasis (Fig. 3B). Consistent with the function of PCK1, gene set enrichment analysis (GSEA) revealed that cellular glucose homeostasis and gluconeogenesis were implicated (Fig. 3C, D). Importantly, the expression of MLK4 and PCK1 appeared to show a tendency of positive correlation in clinical samples (Fig. 3E). PCK1 is one of the isoforms of the phosphoenolpyruvate carboxykinases (PEPCK) and it has another isoform, PCK2. Although PCK2 also appeared to have an oncogenic function in-vitro (Supplementary Fig. 3), we decided to focus on PCK1 because it was enriched by TCGA geneset analysis and had prognostic significance in lung adenocarcinoma, while PCK2 did not.

We further confirmed whether this observed association between MLK4 and PCK1 in clinical samples could be recapitulated in in vitro studies. Knockdown of MLK4 caused a decrease in mRNA expression in *PCK1* (Fig. 3F). The mRNA expression of *PCK1* in lung adenocarcinoma cell lines (Fig. 3G) appeared largely correlated with the expression of *MLK4* (Fig. 3H). When PCK1 was knocked down in lung adenocarcinoma cell lines, a rescue by MLK4 overexpression led to an increase in PCK1 protein expression when compared to PCK1-knockdown cells (Fig. 3I). The inhibitory effects on cell proliferation and colony formation ability were also successfully rescued by MLK4 overexpression (Fig. 3J, K), suggesting that the oncogenic function of MLK4 could be at least partially attributed to PCK1.

Because MLK4 was a kinase, we reasoned that the regulation of PCK1 by MLK4 may be related to the kinase activity of MLK4. Therefore, we further explored the link between MLK4 and PCK1. We turned our attention to CREB, which was well-known for regulating PCK1. Remarkably, MLK4 knockdown reduced the expression level of phosphorylated CREB, and this correlated with the decreased protein expression of PCK1 (Fig. 3L). Immunofluorescence study also demonstrated the decrease in nuclear expression of the phosphorylated (Ser133) form of CREB on MLK4 knockdown (Fig. 3M).

**Fig. 4:**
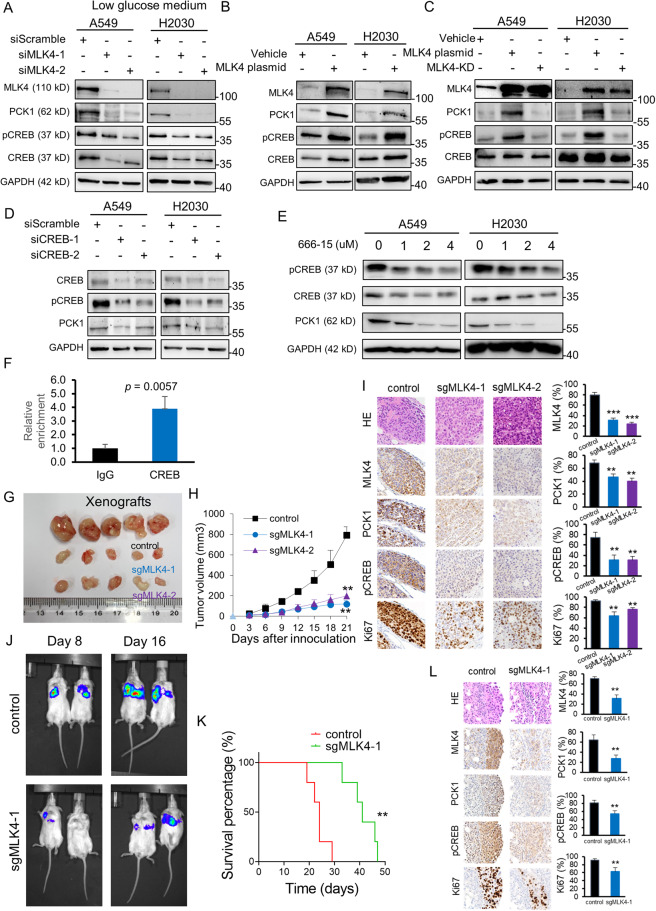
MLK4 influences the expression of PCK1 through phosphorylation of CREB and mediates lung cancer progression in vivo. **A** Western blot analyses demonstrated the reduction of phosphorylated form of CREB and of the expression of PCK1 on MLK4 knockdown in low glucose medium. **B** Overexpression of MLK4 caused an increase in phosphorylated form of CREB and PCK1 expression. **C** Overexpression of kinase dead MLK4 (MLK4-KD) did not lead to the increase in phosphorylated CREB and PCK1 to the extent that was observed in MLK4 wildtype plasmid overexpression. **D** Knockdown of CREB in cancer cell lines resulted in a reduction in PCK1 protein expression. **E** Administration of the pharmacologic inhibitor of CREB-mediated transcription, 666-15, resulted in a reduction of PCK1 protein expression in a dose-dependent manner. **F** Enrichment of CREB on PCK1 promoter in ChIP assay, with antibody towards IgG as control. **G**, **H** Subcutaneous inoculation of MLK4-knocked out cancer cells resulted in a reduced size of A549 xenograft formation in mice, as shown by tumour volume measurements every three days (*n* = 5 for each group, **p < 0.005). **I** Representative photomicrograph images and quantification of MLK4, PCK1, pCREB, and the proliferative index Ki67 in A549 xenografts. 200× magnification. (**p < 0.005; ***p < 0.0005). **J** Orthotopic lung implantation of A549 cancer cells in mice as demonstrated by bioluminescence imaging. **K** Survival analyses of mice (*n* = 5 for each group) which had undergone orthotopic lung implantation of tumour cells. **L** Representative photomicrograph images and quantification of MLK4, PCK1, pCREB, and the proliferative index Ki67 in A549 orthotopically implanted xenografts. 200x magnification. (**p < 0.005).

We were aware that PCK1 in cancer metabolism was most relevant in conditions with scarcity of glucose, therefore, we further performed MLK4 knockdown in cells which were cultured in medium significantly deprived of glucose (Fig. 4A). Importantly, the protein expression of PCK1 was significantly diminished along with a reduction in the phosphorylated form of CREB in siMLK4-transfectants under low glucose culture medium (Fig. 4A). The findings recapitulated those observed in the normal medium condition, and suggested that the MLK4-PCK1 regulatory axis appeared relevant regardless of the relative glucose levels. In contrast, overexpression of MLK4 resulted in a significantly increased level of pCREB and PCK1 (Fig. 4B). Meanwhile, to examine whether the kinase activity of MLK4 was responsible for the phosphorylation of CREB, we utilized the kinase dead variant of MLK4 with the point mutation H261Q. We performed the overexpression of this kinase dead MLK4 in cancer cells. It was observed that while the overall MLK4 expression between the wildtype MLK4 and kinase dead MLK4 groups remained comparable, the expression of the downstream pCREB and PCK1 were lower in the kinase-dead MLK4 transfectants, compared to the wildtype MLK4 group (Fig. 4C). This suggested that the kinase activity of MLK4 was important for the phosphorylation of CREB, and this in turn would lead to PCK1 expression.

To further examine the link of CREB phosphorylation and PCK1 transcription, we performed knockdown and pharmacologic inhibition of CREB in cancer cells. siCREB transfection resulted in a reduction in CREB, pCREB and PCK1 protein expression in the cell lines (Fig. 4D). Furthermore, the administration of 666-15, a selective inhibitor of CREB-mediated gene transcription activity [21, 22], caused a reduction in phosphorylation of CREB as well as the expression of PCK1 in a dose-dependent manner (Fig. 4E). Chromatin immunoprecipitation confirmed considerable enrichment of CREB at the promoter region of PCK1 (Fig. 4F). Taken together, these findings were consistent with the function of CREB as a transcription factor, where CREB phosphorylation was known to mediate PCK1 transcription activation.

Finally, we investigated the functional effect of MLK4 on tumour cells in vivo. Cancer cells were MLK4-knocked out with CRISPR/Cas9 and then inoculated subcutaneously into nude mice. These cells formed significantly smaller tumours than control cells (Fig. 4G, H). Remarkably, immunohistochemical staining of the xenografts showed a decrease of MLK4, pCREB and PCK1 and a decrease in cell proliferation index Ki67 in the sgMLK4 groups (Fig. 4I). In addition, the orthotopic lung implantation mouse model showed a reduction in tumour load and better survival in mice inoculated with MLK4-knocked out cells (Fig. 4J, K). Orthotopically implanted xenografts that were knocked out for MLK4 also showed a reduction in expression of MLK4, PCK1, pCREB, and in the proliferative index Ki67 (Fig. 4L). These in vivo observations reinforced the in vitro findings that MLK4 mediated PCK1 transcription through CREB phosphorylation in lung adenocarcinoma.

### MLK4 promotes enzymatic and metabolic activities of PCK1

**Fig. 5:**
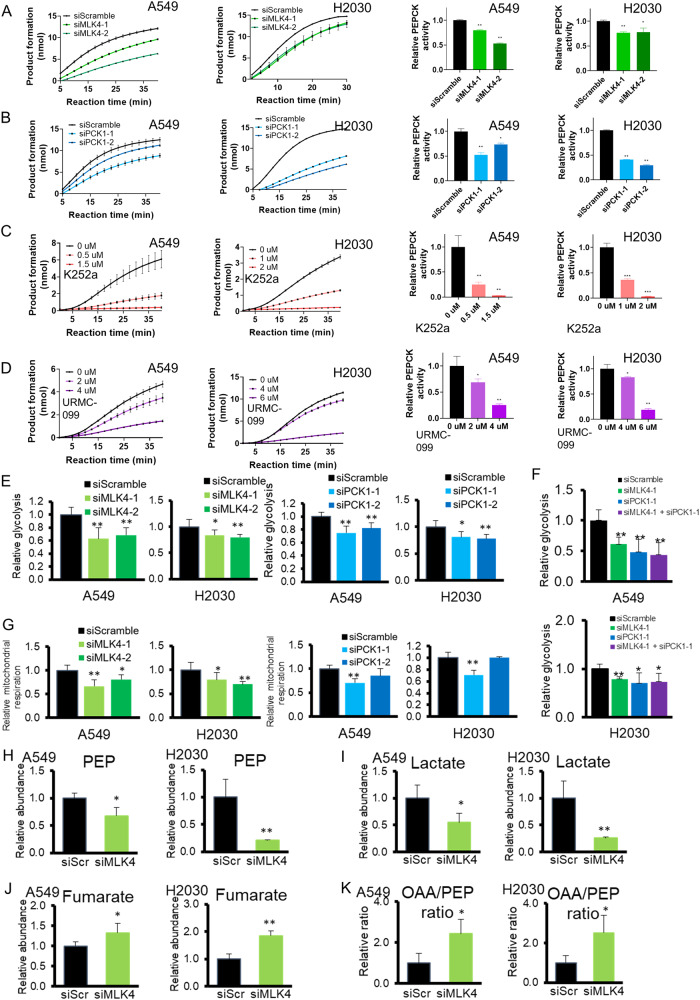
Reduction on phosphoenolpyruvate carboxykinase (PEPCK) activity and the metabolic alteration mediated by depletion of MLK4. **A** siMLK4-transfected A549 and H2030 cells showed a reduction in PEPCK enzymatic activity (*p < 0.05; **p < 0.005). **B** As a positive control, siPCK1 transfectants showed reduction in PEPCK enzymatic activity (*p < 0.05; **p < 0.005). **C**, **D** Administration of the pharmacologic inhibitors of mixed lineage kinase, (**C**) K252a and (**D**) UMRC-099, resulted in a reduction of PEPCK enzymatic activity in a dose-dependent manner. (*p < 0.05; *p < 0.005) **E** Basal glucose metabolic rate in siMLK4 transfectants and siPCK1 transfectants as positive control, demonstrated by the Seahorse metabolic assay (*p < 0.05; **p < 0.005). **F** Glucose metabolic rate of cancer cells with combined siMLK4 and siPCK1 transfections compared to control and single siRNA transfectants, shown by Seahorse assay. (*p < 0.05; **p < 0.005). **G** Mitochondrial respiration capacity measured after the administration of 1.5 μM carbonyl cyanide-4 (trifluoromethoxy) phenylhydrazone (FCCP) in siMLK4 transfectants and siPCK1 transfectants as positive control, using the Seahorse metabolic assay (*p < 0.05; **p < 0.005). **H** Using mass spectrometry, the level of phosphoenolpyruvate (PEP) was shown to be decreased after siMLK4-1 mediated knockdown compared to siScramble (SiScr) (*p < 0.05; **p < 0.005). **I** Lactate, a product of glycolysis, was reduced after siMLK4-1 transfection compared to siScramble (SiScr) (*p < 0.05; **p < 0.005). **J** Fumurate, an intermediate of the TCA cycle, was increased after MLK4 knockdown with siMLK4-1 compared to siScramble (SiScr) (*p < 0.05; **p < 0.005). **K** Oxaloacetate(OAA)-to-PEP ratio was significantly increased on siMLK4-1 mediated knockdown compared to siScramble (SiScr), consistent with a decrease in PEPCK activity (*p < 0.05).

To further support the link between MLK4 and PCK1, we studied if MLK4 could influence the metabolic activities of PCK1. Using a PEPCK activity assay, we found that siMLK4 significantly decreased PEPCK activity (Fig. 5A). To confirm the validity of the finding, we utilized siPCK1 transfectants as a positive control, and noted that the results closely followed the trend observed for siMLK4 transfectants (Fig. 5B). In addition, the administration of pharmacologic inhibitors K252a and URMC-099 with micromolar inhibitory concentration (as determined in Fig. 2L) consistently resulted in a reduction in PEPCK activity in a dose-dependent manner, providing strong evidence that an increased dose of MLK4 inhibitor could result in a stronger inhibition in its downstream effector PCK1 (Fig. 5C, D). We next investigated the effects of MLK4 on glycolysis and mitochondrial respiration. Basal glycolysis was inhibited with MLK4 knockdown (Fig. 5E), which was in keeping with that observed in siPCK1 transfectants. Interestingly, with combined transfections of both siMLK4 and siPCK1, the glycolysis rate of this group closely followed that of the siPCK1 group (Fig. 5F). We reasoned that if the metabolic effect of MLK4 depended on its downstream effector PCK1, in this situation when siPCK1 had already depleted the effector PCK1 molecule to a substantial extent, the effect of knockdown of the upstream MLK4 on PCK1 activity would be diminished. The findings appeared to be consistent with the notion that PCK1 mediated some of the metabolic effect of MLK4.

Meanwhile, the siMLK4-transfected cells also showed a reduction in mitochondrial respiration capacity, measured after the administration of carbonyl cyanide-p-trifluoromethoxyphenylhydrazone (FCCP) (Fig. 5G). siPCK1 transfectants phenocopied the effect of siMLK4 on mitochondrial respiration (Fig. 5G). To understand if the observed changes in metabolic activity translated to an alteration in level of metabolites, we performed mass spectrometry on key intermediates implicated in the enzymatic pathway. PCK1 primarily functions to mediate the conversion of oxaloacetate from TCA cycle to PEP, which in turn could be metabolized into lactate [23]. With the knockdown of MLK4, we observed the reductions in PEP (Fig. 5H) and lactate (Fig. 5I) levels, whereas the levels of TCA cycle metabolite fumarate were increased (Fig. 5J). Moreover, oxaloacetate-to-PEP ratio, a readout inversely correlated to PEPCK activity, was significantly increased (Fig. 5K). The metabolic effects of MLK4 knockdown in lung cancer are therefore consistent with a reduction in PCK1 activity in cancer cells.

### PCK1 exerts an oncogenic effect in lung adenocarcinoma

**Fig. 6:**
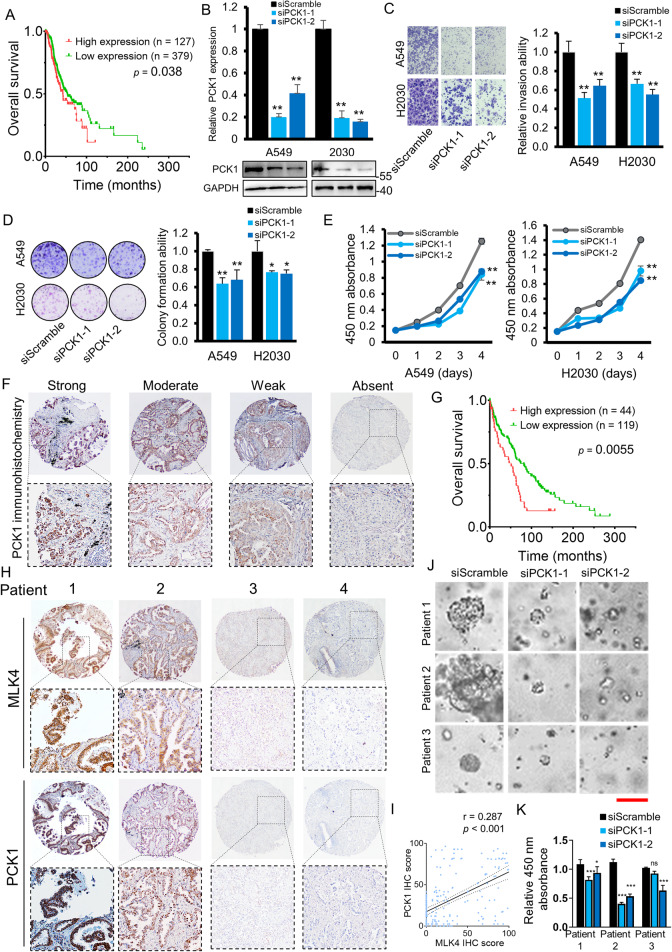
Knockdown of PCK1 exhibits an anti-tumour effect in vitro. **A** PCK1 upregulation is associated with a worse clinical outcome in the Cancer Genome Atlas cohort. **B** The mRNA and protein expression of PCK1 after siRNA-mediated knockdown in A549 and H2030 cells. **C**, **D** Inhibition in (**C**) cell invasion ability by transwell matrigel invasion assay and in the (**D**) monolayer colony formation ability in siPCK1-transfected cells (*p < 0.05; **p < 0.005). **E** siRNA-mediated knockdown of PCK1 inhibited cancer cell proliferation (**p < 0.005). **F** Representative photomicrographs of PCK1 immunohistochemistry (IHC) on tissue microarray, showing cytoplasmic staining in cancer cells. 40× (above) and 100× magnification (below). **G** High protein expression of PCK1 as indicated by strong or moderate IHC staining was associated with a worse patient outcome. **H** Representative photomicrographs for immunohistochemistry with strong/moderate or negative expression of MLK4 and PCK1. 40× (above) and 100× magnification (below). **I** Correlation of MLK4 and PCK1 IHC scores in lung adenocarcinoma tissue microarrays. **J**, **K** (**J**) Representative photomicrographs of the inhibition in the growth of patient-derived organoids treated with siPCK1, and (**K**) quantification with the cell counting kit under 450 nm absorbance. Scale bar, 50 μm (*p < 0.05; **p < 0.005; ***p < 0.0005).

PCK1 catalyzes the key step of oxaloacetate to PEP conversion. After showing the possible link between MLK4 and PCK1, we sought to establish the role and function of PCK1 in carcinogenesis. Clinically, a high expression of PCK1 portended a poorer survival in lung adenocarcinoma (Fig. 6A). After the knockdown of PCK1 using siRNA (Fig. 6B), cell invasion and colony formation (Fig. 6C, D), as well as cell growth (Fig. 6E) abilities of the tumour cells, were all decreased. Immunohistochemical studies on lung adenocarcinoma tissue microarray demonstrated that patients with high PCK1 expression had a worse prognosis, compatible to the findings in literature (Fig. 6F, G). A positive correlation between MLK4 and PCK1 was also suggested in tissue microarray clinical cohorts (Fig. 6H, I). To evaluate whether the survival difference for lung adenocarcinoma patients with high PCK1 level could be accounted for by using in vitro model, we performed knockdown of PCK1 in patient-derived organoids, which also led to suppression of organoid growth (Fig. 6J, K). Taken together, these findings demonstrated that PCK1 exerts an oncogenic effect in lung adenocarcinoma.

### KLF5 regulates MLK4 expression and promotes lung adenocarcinoma progression

**Fig. 7:**
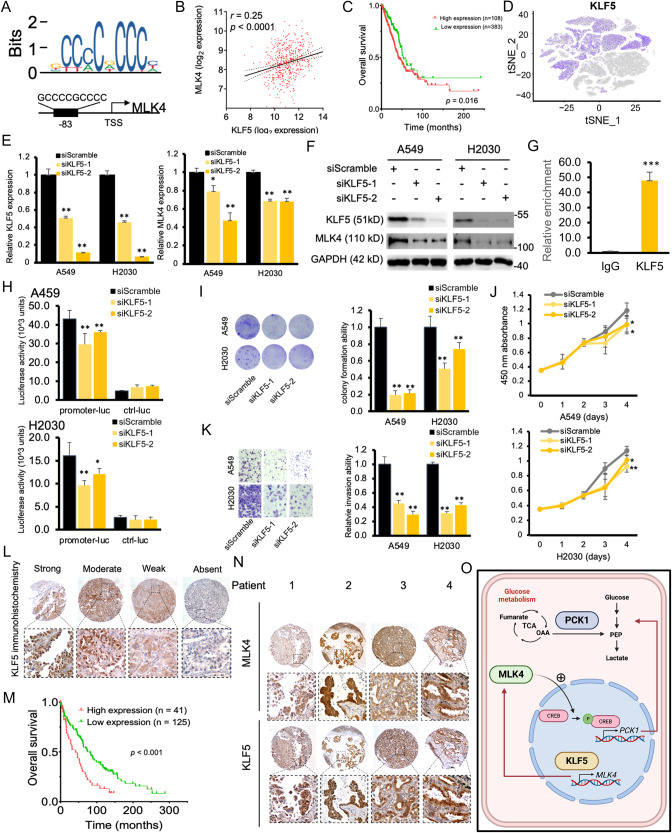
Identification of KLF5 as the transcription factor of MLK4 and the oncogenic activity of KLF5 in vitro. **A** The putative transcription factor binding site at the promoter of MLK4. TSS, transcription start site. **B** KLF5 mRNA expression showed a tendency to be correlated with that of MLK4 in the Cancer Genome Atlas (TCGA) cohort. **C** KLF5 upregulation was associated with a worse clinical outcome in TCGA cohort. **D** The expression of KLF5 in various cell types in lung adenocarcinoma from single cell RNA-seq analysis, where the colour code was provided in Fig. 1E. **E** Knockdown of KLF5 resulted in a reduction in mRNA expression in MLK4 (*p < 0.05; **p < 0.005). **F** Western blot showed the reduction of MLK4 protein expression with KLF5 knockdown. **G** Enrichment of KLF5 on MLK4 promoter in ChIP assay, when using antibody against IgG as control. **H** SiKLF5 caused a significant reduction in luciferase reporter activity in constructs encompassing the promoter of MLK4 (promoter-luc), compared to the constructs containing control sequences (ctrl-luc). **I** Knocking down KLF5 resulted in a reduction of monolayer colony formation ability of the cancer cells (**p < 0.005). **J** siKLF5 tranfectants had a reduced cell proliferation rate (*p < 0.05; **p < 0.005). **K** KLF5 knockdown inhibited cell invasion ability in transwell matrigel invasion assay (**p < 0.005). **L** Representative photomicrographs of KLF5 immunohistochemistry (IHC) on tissue microarray, showing nuclear staining in cancer cells. **M** High protein expression of KLF5 as noted by strong or moderate IHC staining was associated with a worse patient outcome. **N** Representative photomicrographs for immunohistochemistry of MLK4 and KLF5 on lung adenocarcinoma tissue microarray, with micropapillary (#1), papillary (#2) and acinar (#3 and 4) invasion patterns. **O** Schematic figure showing the link between the key signaling molecules in the oncogenic and metabolic cascade. KLF5 is a transcription factor and causes the expression of MLK4. As a kinase, MLK4 either directly or indirectly phosphorylates the transcription factors CREB, which subsequently binds to the promoter of PCK1, leading to its protein expression. The PCK1 enzyme catalyzes the rate-limiting step of oxaloacetate (OAA) in the tricarboxylic acid cycle (TCA) to phosphoenolpyruvate (PEP), which is an intermediate for glucose metabolism of cancer cells. Plus sign indicates an activation effect.

To investigate if transcriptional events may be implicated in MLK4 upregulation other than copy number alterations (Fig. 1D), we studied the MLK4 promoter with computational analyses to evaluate its possible transcription factors. From JASPAR database, the transcription factor KLF5 ranked high in the interaction with the MLK4 promoter and its putative binding site started 83 base pairs proximal to the transcription start site (Fig. 7A). In clinical samples, expression of KLF5 also correlated with that of MLK4 (Fig. 7B). In the TCGA cohort, KLF5 upregulation led to a worse prognosis (Fig. 7C). Its expression was predominantly increased in cancer cells (Figs. 7D, 1E), and a subset of these highly expressed KLF5 cells appeared to coincide with those cells having relatively high MLK4 expression (Figs. 7D, 1F).

To validate the association between KLF5 and MLK4, we performed KLF5 knockdown. As shown in Fig. 7E, F, siKLF5 reduced both the mRNA and protein expression of MLK4. To confirm the direct involvement of KLF5 in mediating transcription of MLK4, we performed ChIP-qPCR assay and cloned the MLK4 promoter into a luciferase reporter plasmid. Consistent with our hypothesis, significant enrichment of KLF5 on MLK4 promoter was found in ChIP study (Fig. 7G), while luciferase activities were significantly decreased in siKLF5-transfected cells (Fig. 7H). The oncogenic function of KLF5 was further demonstrated by means of colony formation (Fig. 7I), cell proliferation (Fig. 7J), as well as invasion assays (Fig. 7K). Immunohistochemistry on tissue microarray revealed nuclear staining for KLF5 antibody, in keeping with its role as a transcription factor (Fig. 7L). A minority of cases showed strong or moderate staining for KLF5, and these patients appeared to have a worse survival (Fig. 7M). Co-expression of KLF5 and MLK4 were observed in lung adenocarcinoma with diverse patterns of invasion (Fig. 7N).

## Discussion

Whether MLK4 is an oncogene is controversial, and its action appears to be cancer-type specific. It is currently unknown whether MLK4 is an oncogenic factor for lung cancer, and what upstream and downstream signaling molecules are involved. Our study demonstrated that the KLF5-MLK4-PCK1 axis exerts an oncogenic function in lung carcinogenesis with altered glucose metabolism. These findings are interesting in that we unraveled the unusual link between a kinase chiefly involved in the cell cycle progression and a metabolic enzyme [24]. Moreover, we showed that MLK4 and PCK1 are oncogenic factors in lung cancer with potential clinical and prognostic significance.

We revealed that MLK4 activates PCK1 expression via CREB. PCK1 was known in the literature to demonstrate a generally oncogenic function [25, 26], and the cytosolic and mitochondrial isoforms of PEPCK, named PCK1 and PCK2 respectively, appear to have comparable functions in existing studies [26]. Here, we showed that PCK1 exerts oncogenic function in lung cancer. Notably, PCK1 protein expression is high in lung cancer cell lines, and its knockdown also mediated prominent inhibitory effects in cancer cell growth, invasion and colony formation. Our study thus sheds light on the oncogenic role of PCK1 in lung cancer and its enzymatic function in modulating glucose metabolism.

PCK1 mainly catalyzes the phosphorylation and decarboxylation of oxaloacetate and produces PEP, which is known to be a rate-limiting step between the tricarboxylic acid cycle and the glucose metabolism pathway [27]. Since this is a predominantly unidirectional process, blockade of the MLK4-PCK1 axis might affect glucose metabolism. Consistent with this notion, we found that MLK4 knockdown reduced basal glycolysis and mitochondrial respiration capacity. In addition, we measured metabolite flux by LC-MS, revealing that PEP and lactate, the downstream metabolites of PEPCK, were down-regulated by the knockdown of MLK4. Overall, the findings are compatible with the proposal that MLK4 is an upstream regulator of PCK1, and it mediates its metabolic effect on cancer cells through the transcriptional activation of PCK1. This finding is also entirely consistent with the known effect on glucose metabolism alteration, including reduced glycolysis, on PEPCK inhibition [26].

Although PCK1 expression in tumours may not be very high in some publicly-available database such as the Human protein atlas, it must be acknowledged that PCK1 expression can be dynamic in the cells and can become increasingly expressed when the tumour experiences a low glucose condition. We reason that it is an important oncogenic event responsible for the downstream effects of MLK4. We demonstrated that PCK1 expression could be high in a significant subset of patients using immunohistochemistry on tissue microarray, that it was significantly correlated with a worse prognosis, and that it was regulated by MLK4 and CREB in low glucose media conditions. Meanwhile, CREB was well-known to regulate the transcription of PCK1, at least in gluconeogenic tissues [24, 28]. By siRNA-mediated CREB inhibition and selective pharmacologic inhibitor of CREB, we demonstrated that this regulation of PCK1 by CREB was also present in lung adenocarcinoma.

The knockdown of MLK4 and PCK1 has significantly suppressed glycolysis, and as expected has not fully inhibited glycolysis. This is compatible to the Warburg effect [29, 30], such that since the cancer cells heavily rely on aerobic glycolysis for survival, compensatory mechanisms may have been in place to overcome the reduction of glycolysis caused by MLK4 or PCK1 knockdown. While the oncogenic functions were demonstrated by knockdown of MLK4 in vitro, and a metabolic alteration was also observed, it was not certain whether other non-metabolic mechanisms or signaling pathways also mediated the oncogenic properties of MLK4, and this will require future investigations.

We also examined the mechanism underlying the overexpression of MLK4 in lung cancer. We uncovered that the KLF5 transcriptional factor might be important in its regulation, as evidenced by in silico prediction and in vitro luciferase reporter assay. Knockdown studies validated the direct regulation of MLK4 by KLF5. Our results were strengthened by clinical data, as KLF5 expression positively correlated with that of MLK4 from immunohistochemistry on tissue microarrays and TCGA dataset. Single cell sequencing analysis also demonstrated that a subset of lung cancer cells with high KLF5 expression has a concomitant elevation of MLK4. Besides, KLF5 and MLK4 upregulation in lung adenocarcinoma led to a worse prognosis, verifying the importance of KLF5-MLK4 axis in lung cancer.

Taken together, a model of oncogenic KLF5-MLK4-PCK1 pathway was elucidated (Fig. 7O). Transcription factor KLF5 binds to the MLK4 promoter, inducing its transcription. Subsequently, MLK4 directly or indirectly phosphorylates transcription factor CREB, causing the transcriptional activation of PCK1 [28, 31–33]. The expression of PCK1 leads to an increase in their enzymatic activities and a conversation of oxaloacetate from the tricarboxylic acid cycle to the glucose metabolic pathway, and subsequently enhances glucose metabolism that supports increased cell proliferation and tumorigenesis.

In summary, our study has uncovered several molecules with previously unascertained oncogenic function in lung adenocarcinoma, constituting a signaling pathway governing glucose metabolism in cancer cells. The MLK4 kinase that is central to this pathway was previously thought to be involved in the classical pathways for cell cycle progression and inflammatory response, but has been found to be able to modulate the expression of a metabolic enzyme in our study, by mediating the phosphorylation of the transcription factor CREB. As many targeted therapy agents in lung cancer are kinase inhibitors [34], the discovery of novel therapeutic agents will undoubtedly be aided by a better understanding of the diverse phosphorylome of kinases.

## Supplementary information

Supp

## Data Availability

The data that support the findings of this study are available from the corresponding author upon reasonable request. Bioinformatics analyses performed on TCGA datasets can be retrieved from cBioportal and TCGA repository. No other large scale omics datasets have been involved in the generation of the study results.
