# Supplementary material for: MLK4 promotes glucose metabolism in lung adenocarcinoma through CREB-mediated activation of phosphoenolpyruvate carboxykinase and is regulated by KLF5
Source: Oncogenesis. 2023 Jul 5;12(1):35. doi: 10.1038/s41389-023-00478-y (PMC10323122; doi:10.1038/s41389-023-00478-y)
Supplement: Supplementary file 1 — Supp [file 41389_2023_478_MOESM1_ESM.docx]

**Supplementary Information**

**MLK4 promotes glucose metabolism in lung adenocarcinoma through CREB-mediated activation of phosphoenolpyruvate carboxykinase and is regulated by KLF5**

Alvin Ho-Kwan Cheung, Kit-Yee Wong, Xiaoli Liu, Fenfen Ji , Chris Ho-Lam Hui, Yihan Zhang, Johnny Sheung-Him Kwan, Bonan Chen, Yujuan Dong, Raymond Wai-Ming Lung, Jun Yu, Kwok Wai Lo, Chi Chun Wong, Wei Kang, Ka Fai To

**Content:**

1. **Supplementary Figures**
2. **Supplementary Table**

**Supplementary Figure 1**

**
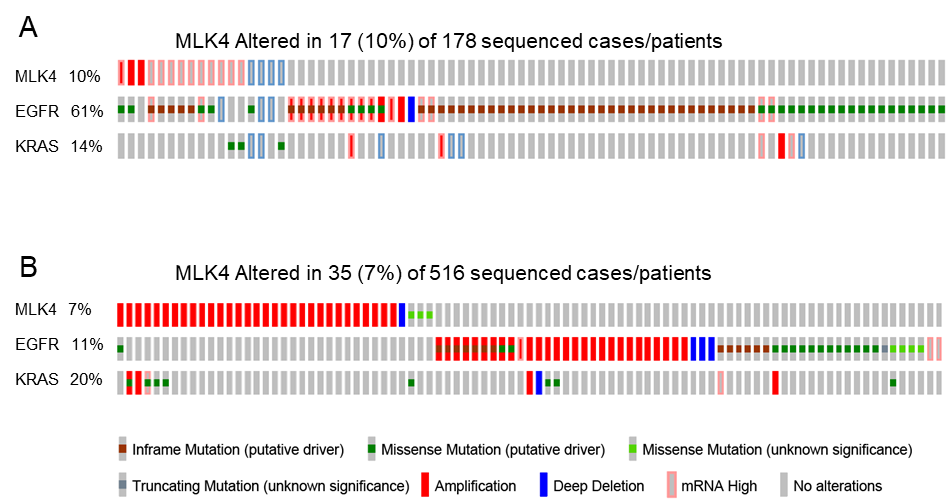
**

**Figure S1. MLK4 alteration occurs in a significant subset of lung adenocarcinoma patients.** Data from cbioportal for the (A) Lung Adenocarcinoma OncoSG cohort, 2020 and (B) Lung Adenocarcinoma TCGA, Firehose Legacy cohort. Note that data from the TCGA PanCancer Atlas was shown in Figure 1A, while the other TCGA cohorts available on cbioportal contained overlapping samples and thus were not analyzed in combination.

**Supplementary Figure 2**


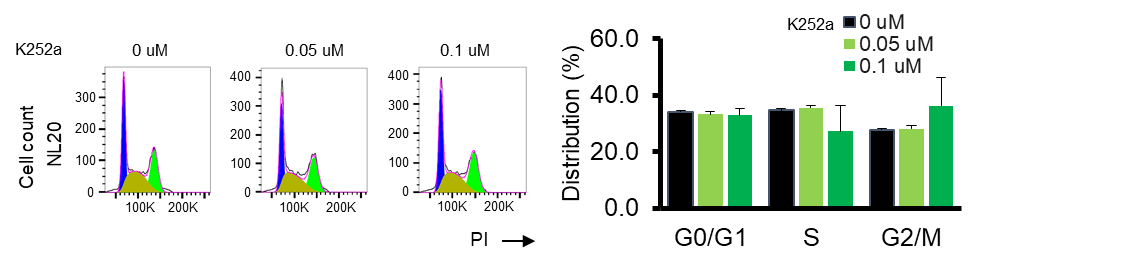


**Figure S2**. FACS analysis for the cell cycle distribution in NL20 treated with K252a.

**Supplementary Figure 3**

**
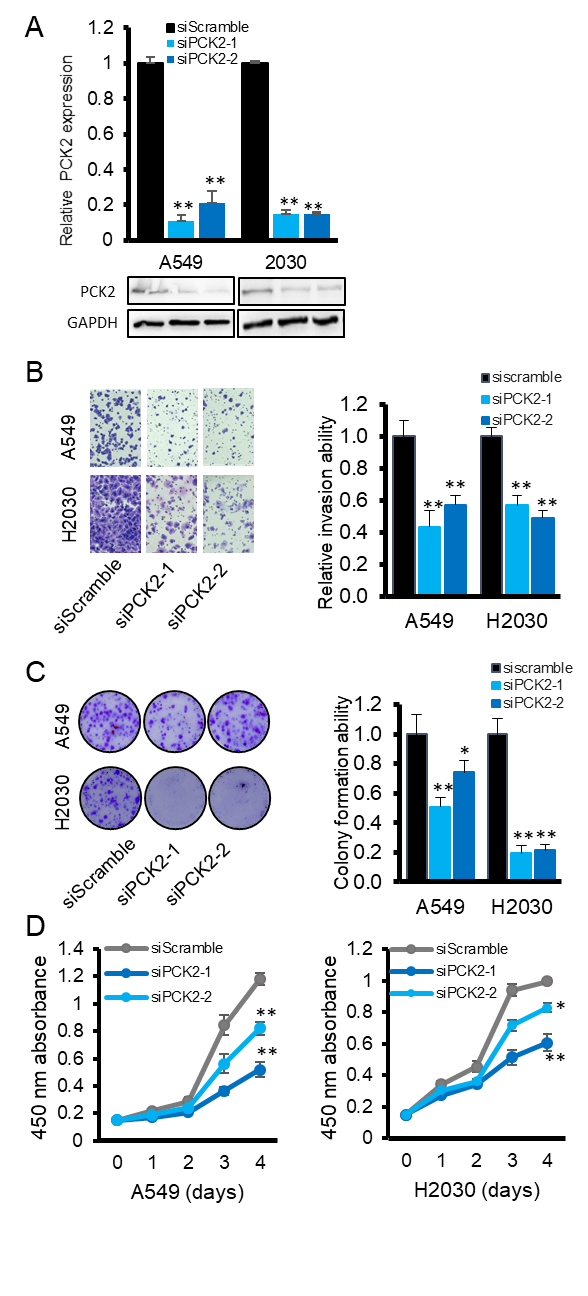
**

**Figure S3.** PCK2 exerts an oncogenic function in lung adenocarcinoma. A. The mRNA and protein expression of PCK2 after siRNA-mediated knockdown in A549 and H2030 cells. B, C. Reduction in (B) cell invasion ability and (C) monolayer colony formation ability in siPCK2-transfected cells (*, p < 0.05; **, p < 0.005). (D) siRNA-mediated knockdown of PCK2 inhibited cancer cell proliferation (**, p < 0.005).

**Supplementary Table 1.** Nucleic acid sequences in this study.

|  | Primers for quantitative PCR |
| --- | --- |
| MLK4-F | CTTTGCCCATTCCATCCACC |
| MLK4-R | CGCAGCTCCTTTTCCTTTGT |
| PCK1-F | CACATGCTGATTCTGGGTATAAC |
| PCK1-R | CATCCAGGCAATGTCATCCC |
| PCK2-F | GGCTGAGAATACTGCCACACT |
| PCK2-R | ACCGTCTTGCTCTCTACTCGT |
| KLF5-F | TGGTCCAGACAAGATGTGAAA |
| KLF5-R | CAGGTGAGTGATGTCAGGGA |
| GAPDH-F | GTCTCCTCTGACTTCAACAGCG |
| GAPDH-R | ACCACCCTGTTGCTGTAGCCAA |
|  |  |
|  | Target DNA sequence of sgRNAs |
| sgMLK4-1 | AGCCTCGTAGTCATAGAGCG |
| sgMLK4-2 | CTCTATGACTACGAGGCTCG |
|  |  |
|  | Primers for ChIP-qPCR |
| MLK4-F | GCTCACCATTGCTCCGGAG |
| MLK4-R | TTCATTCCGCCCTCCTTGAC |
| PCK1-F | CAGTAAAATGGGTCAAGGTT |
| PCK1-R | GTTATGCTTTGGGCCAACTC |
|  |  |
|  | Insert for luciferase reporter assay |
| Promoter-luc | GCACCTGCGGCGGGGGCGGGGTGGGACCAGCCAGGCGGGCCCAGGGGGCGGGCCCGGCCCAAGGTGGTGGGGGACGGGCCCGGGCGCCAGGGGCGGGACGAGGCTCTGGGGACTGGCACAGAAAAGGGAAGTGAACCTGGCGAGGGCGGGGCGGGATGGTGCAGTTGGTGGGGAAGGGTGGGGCGAACCTAGGAAGGCGGTCCCATAGGGCGGGCGCAGACTGGGCGCCCGGGACGGGTCAAGGAGGGCGGAATGAACCTGGAGAGGGCGGGGCGGCTGGGGGAGGGGCGGGAAGGCCCTGGGGACTGGCCTAGGGGCGGGCCCGGGGGGGTGGGGGGGCGGTGCGGAATGGAGCCGGAGGGGGCGGGGCGGCCCTGGCGCGGCTAGGGTGGGGTGGGGC |
| Control-luc | TAAAGTAGGTACTATTTATTATATTCCCATTTTATAGATGAGAAAACTGAGTCATAGAGAAATAAAGAAACTTGCTCAAGGTCACACAGCCAATAAATAGTTTGATCTGAGAGACAAAGAAAACTGAAAAGACAATAAATTTATCTTTATGTAACTCAGAATGTTACTGTATGTCCTATCCTCATAATATCTAAAATTGTCCCTTCTGTTATTGGAAGTACAGGATCCACACGTTAGGAAATTCTGGCCTACTACAGGATAATCTAATATTTTAGAATTTTAATAACTAAGCTCTTCTATCTTTTTAGCCTCATCACCTGCCACAGTAACCTACCAACGTCTGGCACCCAGCCATTGCCAATGCTTCCTAAACCCAAACCTGTGGGTTTCCAGGGAGACT |
